# Potassium/Sodium Citrate Attenuates Paclitaxel-Induced Peripheral Neuropathy

**DOI:** 10.3390/ijms26073329

**Published:** 2025-04-03

**Authors:** Daisuke Uta, Hideki Nakamura, Kengo Maruo, Kanoko Matsumura, Yohei Usami, Toshiaki Kume

**Affiliations:** 1 Department of Applied Pharmacology, Faculty of Pharmaceutical Sciences, University of Toyama, Toyama 930-0194, Japan; tkume@pha.u-toyama.ac.jp; 2 Discovery Research Laboratories, Nippon Chemiphar Co., Ltd., Saitama 341-0005, Japan; h-nakamura@chemiphar.co.jp (H.N.); k-maruo@chemiphar.co.jp (K.M.); k-matsumura@chemiphar.co.jp (K.M.); y-usami@chemiphar.co.jp (Y.U.)

**Keywords:** pain, paclitaxel, mechanical allodynia, alkalinization, potassium/sodium citrate, electrophysiology, spinal dorsal horn

## Abstract

Chemotherapy-induced peripheral neuropathy (CIPN) is a significant adverse event with unclear mechanisms and limited treatment alternatives. This study aimed to investigate the efficacy of two alkalizing agents, a mixture of potassium citrate and sodium citrate (K/Na citrate) or sodium bicarbonate (NaHCO_3_), in preventing and treating paclitaxel (PTX)-induced mechanical allodynia in rodents. The results from rodent models demonstrated that repeated prophylactic administration of K/Na citrate or NaHCO_3_ could inhibit the development of PTX-induced mechanical allodynia. Moreover, K/Na citrate was effective in preventing the PTX-induced exacerbation of mechanical allodynia, even when treatment was initiated immediately after the onset of allodynia. K/Na citrate also reduced the levels of the plasma complement component anaphylatoxin C3a in a PTX-induced CIPN rat model. Complement activation, resulting in the production of C3a, has been implicated in the pathogenesis of this model. Additionally, pretreatment with Na citrate significantly prevented the reduction in neurite outgrowth caused by PTX. Furthermore, K/Na citrate inhibited spontaneous and mechanical stimuli-induced firing in spinal dorsal horn neurons. These findings indicate that K/Na citrate may regulate the development of PTX-induced mechanical allodynia by modulating complement activation and providing neuroprotection against PTX-induced peripheral nerve injury. This study implies that alkalization could help prevent PTX-induced peripheral neuropathy and mitigate its exacerbation.

## 1. Introduction

Most cancers can be treated with anticancer drugs, such as platinum-based drugs like oxaliplatin, taxanes like paclitaxel (PTX), vinca alkaloids like vincristine, and proteasome holoenzyme inhibitors like bortezomib. However, these drugs can induce peripheral neuropathy [1,2,3,4].

Chemotherapy-induced peripheral neuropathy (CIPN) can present with sensory neuropathy symptoms, such as numbness, hypoesthesia, and pain in the extremities; motor neuropathy symptoms, such as muscular atrophy, weakness, and flaccid paralysis with impaired intestinal motility and involuntary muscles, dysuria, dyshidrosis, and orthostatic hypotension; and autonomic neuropathy symptoms, such as constipation. PTX, in particular, is associated with sensory and motor neuropathy [5]. CIPN can also present as shooting or burning pain that can be persistent and debilitating, having a significant impact on patients’ quality of life.

CIPN is a well-known adverse event; however, the detailed mechanism underlying its development remains unclear, resulting in a lack of established effective treatment alternatives. CIPN significantly impacts the continuation of cancer treatment; hence, its prevention and treatment are crucial for improving treatment outcomes and enhancing patients’ quality of life. Currently, the discontinuation of anticancer drugs is the only available option to alleviate CIPN symptoms [6].

In terms of CIPN prevention, calcium or magnesium may be administered to prevent neurotoxicity; however, there is no clear evidence to support their effectiveness. Drugs commonly used to manage CIPN-associated numbness or pain may include *Goshajinkigan*, vitamin B12 preparations, nonsteroidal anti-inflammatory drugs, pregabalin, gabapentin, and opioids. However, there is a lack of evidence behind the recommended specific dosages and their efficacy remains unclear [7,8,9,10,11,12,13].

CIPN’s pathogenesis is multifaceted, with immune cell-mediated neuroinflammation involving microglia, astrocytes, and macrophages playing a significant role [14,15,16]. A previous study suggested that a decrease in the local pH around neurons at CIPN’s onset is a contributing factor, as astrocyte activation can result in excessive lactic acid production [17]. In addition, allodynia may be induced by the excessive excitation of nerve cells.

PTX and oxaliplatin have been reported to decrease the velocity of the peripheral blood flow [18], suggesting that the peripheral neuropathy induced by these drugs contributes to sensory disturbances such as pain and numbness.

Numbness is an unpleasant sensation that can occur spontaneously or in response to external stimuli, often accompanied by pain, paresthesia, hypoesthesia, and a loss of sensation. Although the exact mechanism underlying numbness remains to be completely understood, ischemia–reperfusion resulting from blood flow occlusion and restoration is expected to be involved.

An inadequate oxygen supply, either due to insufficient blood flow or ischemia–reperfusion, can enhance the glycolytic pathway, thus decreasing the pH [19,20,21,22]. Therefore, the local pH may decrease at sites with inadequate blood flow. Such decreases in local pH may occur in the peripheral nerves at CIPN’s onset. However, the effectiveness of alkalization in CIPN remains unknown.

PTX can enhance the therapeutic effect of anticancer agents in pancreatic cancer [23], and the efficacy of alkalinization therapy in oncology has attracted attention. PTX is a standard anticancer drug used in the treatment of pancreatic cancer [24,25]. Thus, evaluating the effect of alkalinization on the development of PTX-induced CIPN is important.

Therefore, this study aimed to investigate the efficacy of alkalization in PTX-induced CIPN.

## 2. Results

### 2.1. Preventive Effect of Repeated Oral Administration of a Mixture of Potassium Citrate and Sodium Citrate (K/Na Citrate) on Mechanical Allodynia Induced by PTX in Mice

**Figure 1:**
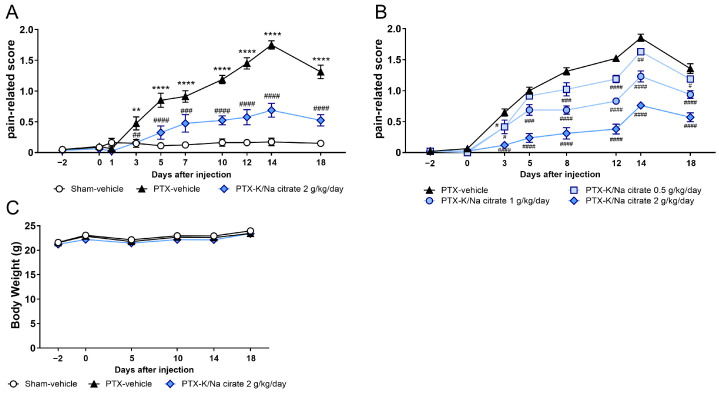
Preventive effect of repeated K/Na citrate oral administration on PTX-induced mechanical allodynia in mice. (**A**,**B**) Time course of the pain-related scores in PTX-treated mice orally administered K/Na citrate or without K/Na citrate at doses of 2 g/kg/day (**A**) or at 0.5, 1, or 2 g/kg/day (**B**). (**C**) Body weights of mice from each group in (**A**). Data are presented as mean ± standard error of the mean (SEM) (*n* = 7–8). ** *p* < 0.01, **** *p* < 0.0001 vs. sham-vehicle. ^#^ *p* < 0.05, ^##^ *p* < 0.01, ^###^ *p* < 0.001, ^####^ *p* < 0.0001 vs. PTX-vehicle (two-way repeated measures analysis of variance (ANOVA) with post hoc Holm–Sidak multiple comparison test).

We investigated the potential preventive effects of the alkalinizing agent K/Na citrate on PTX-induced mechanical allodynia in mice. A single injection of PTX (5 mg/kg; intraperitoneal, i.p.) caused a significant increase in pain-related scores compared with those of sham mice from day 3 until day 16, which peaked on day 14 (Figure 1A, day 14, 0.18 ± 0.06, sham-vehicle group; 1.75 ± 0.07, PTX-vehicle group, *p* < 0.0001). The repeated administration of K/Na citrate (2 g/kg/day; per os, p.o.) significantly suppressed PTX-induced mechanical allodynia on days 3, 5, 7, 10, 12, 14, and 18 (Figure 1A, day 14, 0.69 ± 0.11, PTX-K/Na citrate group, *p* < 0.0001). This antiallodynic effect of K/Na citrate was found to be dose-dependent over the course of PTX-induced allodynia (Figure 1B). PTX injection or K/Na citrate administration did not alter the body weights of mice (Figure 1C). In addition, previous studies have demonstrated that K/Na citrate does not have a sedative effect [26]. Therefore, the observed analgesic effect of K/Na citrate is unlikely to be attributed to sedation or the body weight loss induced by its administration. Thus, in subsequent experiments, the dose of 2 g/kg/day, which most effectively suppressed mechanical allodynia, was employed.

### 2.2. Blood Bicarbonate (HCO_3_^−^) Concentrations and pH After the Administration of K/Na Citrate

**Figure 2:**
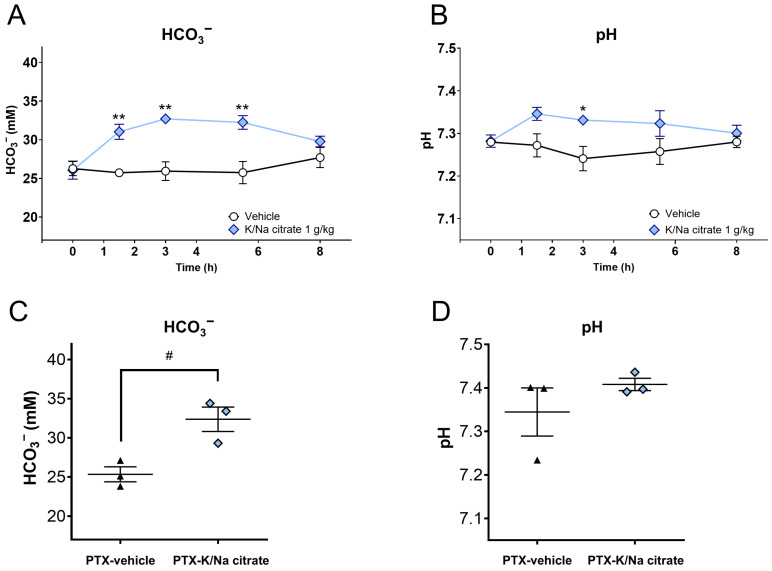
Blood HCO_3_^−^ concentrations and pH after oral administration of K/Na citrate. (**A**,**B**) Blood HCO_3_^−^ concentrations (**A**) and blood pH (**B**) in healthy C57BL/6 mice after a single administration of K/Na citrate (1 g/kg, p.o.), measured at different time points post administration (0, 1.5, 3, 5.5, and 8 h). Data are presented as mean ± SEM (*n* = 3–4). * *p* < 0.05, ** *p* < 0.01 vs. vehicle (two-way ANOVA with post hoc Holm–Sidak multiple comparison test). (**C**,**D**) Blood HCO_3_^−^ concentrations (**C**) and pH (**D**) in PTX-induced CIPN mice after repeated administration of K/Na citrate (2 g/kg/day, p.o.), measured on day 14 at 2 h post administration. Data are presented as mean ± SEM (*n* = 3). ^#^ *p* < 0.05 vs. PTX-vehicle (Student’s *t*-test).

We investigated whether K/Na citrate has an alkalinization effect in the PTX mouse model that is similar to that in naive mice. In naive mice, an increase in blood HCO_3_^−^ concentration was observed after the oral administration of K/Na citrate, leading to a concomitant increase in blood pH. In particular, a single oral dose of K/Na citrate significantly increased the blood’s HCO_3_^−^ concentration (Figure 2A, at 1.5, 3, and 5.5 h, *p* < 0.01) and pH (Figure 2B, 3 h, *p* < 0.05) in naive mice. In the PTX-induced CIPN mice, the repeated oral administration of K/Na citrate also increased blood HCO_3_^−^ concentrations on day 14 at 2 h after the administration of K/Na citrate (Figure 2C, *p* < 0.05); however, it did not upregulate the blood pH significantly (Figure 2D).

### 2.3. Comparison of the Effect of Repeated Oral Administration of K/Na Citrate and Sodium Bicarbonate (NaHCO_3_) on PTX-Induced Mechanical Allodynia

**Figure 3:**
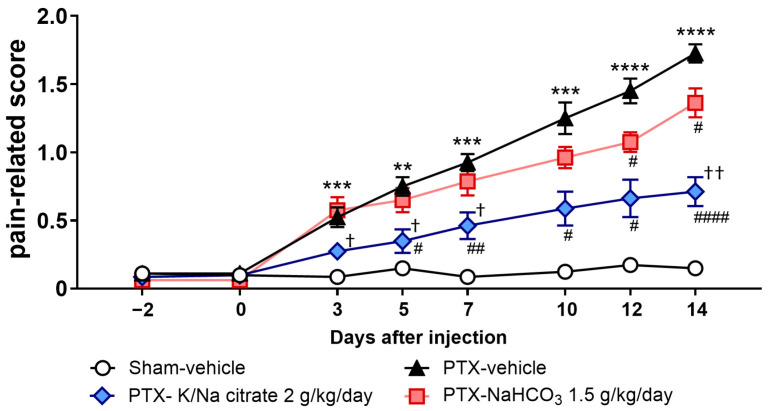
Comparison of the effects of repeated oral administration of K/Na citrate and NaHCO_3_ on PTX-induced mechanical allodynia. Time course of the pain-related scores in PTX-treated mice administered K/Na citrate (2 g/kg/day, p.o.) or NaHCO_3_ (1.5 g/kg/day, p.o.) and those without either administered. Data are presented as mean ± SEM (*n* = 8). ** *p* < 0.01, *** *p* < 0.001, **** *p* < 0.0001 vs. sham-vehicle. ^#^ *p* < 0.05, ^##^ *p* < 0.01, ^####^ *p* < 0.0001 vs. PTX-vehicle. ^†^ *p* < 0.05, ^††^ *p* < 0.01 vs. PTX NaHCO_3_ (two-way repeated measures ANOVA with post hoc Holm–Sidak multiple comparison test).

The antiallodynic effect of K/Na citrate may be mediated through the upregulation of blood HCO_3_^−^. Therefore, we investigated the effect of NaHCO_3_ at a dose of 1.5 g/kg/day, which has an equivalent blood alkalizing effect to K/Na citrate at 2 g/kg/day, on PTX-induced mechanical allodynia as a comparative study. NaHCO_3_ also suppressed PTX-induced mechanical allodynia (Figure 3, day 14, 1.36 ± 0.11, PTX-NaHCO_3_ group; 1.73 ± 0.07, PTX-vehicle group, *p* < 0.05); however, the antiallodynic effect of NaHCO_3_ was significantly weaker than that of K/Na citrate (Figure 3, day 14, 0.71 ± 0.11, PTX-K/Na citrate group, *p* < 0.01).

### 2.4. Effect of Repeated Oral Administration of K/Na Citrate on Spontaneous Firing and Von Frey Filament (vFF)-Evoked Firing Induced by PTX in Superficial Dorsal Horn Neurons

**Figure 4:**
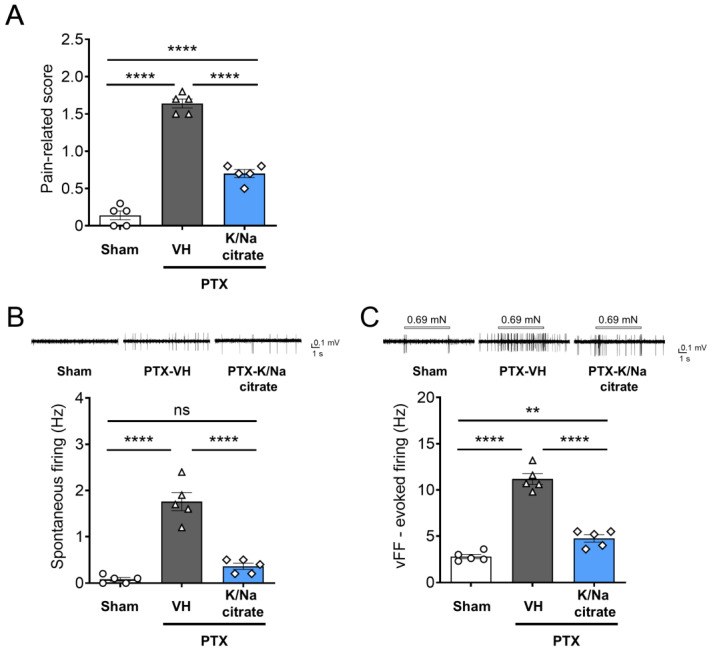
Effect of repeated oral administration of K/Na citrate on spontaneous firing and vFF-evoked firing in superficial dorsal horn neurons in PTX-treated mice. (**A**) Pain-related scores in PTX-induced CIPN model mice with or without K/Na citrate treatment on day 13. (**B**,**C**) Representative traces and quantitative evaluation of spontaneous firing (**B**) and vFF-evoked firing (**C**) in superficial dorsal horn neurons in mice receiving each treatment. Data are presented as mean ± SEM (*n* = 5) (the total number of neurons was 25). ** *p* < 0.01, **** *p* < 0.0001 (one-way ANOVA with post hoc Holm–Sidak multiple comparison test).

On day 13, the pain-related scores increased with the PTX treatment (Figure 4A, day 13, 1.64 ± 0.06, PTX-vehicle group; 0.14 ± 0.06, sham-vehicle group; *p* < 0.0001) and reduced with K/Na citrate administration (Figure 4A, day 13, 0.70 ± 0.05, PTX-K/Na citrate group, *p* < 0.0001), consistent with the abovementioned results. Spontaneous firing in superficial dorsal horn neurons was significantly elevated in PTX-treated mice but not in those treated with the vehicle only on day 14 (Figure 4B, day 14, 1.76 ± 0.20, PTX-vehicle group; 0.08 ± 0.04, sham-vehicle group, *p* < 0.0001). The repeated administration of K/Na citrate (2 g/kg/day, p.o.) significantly attenuated this effect by 80% (Figure 4B, day 14, 0.36 ± 0.07, PTX-K/Na citrate group, *p* < 0.0001). Mechanical stimuli-induced firing with the vFF elicited transient firing in the superficial dorsal horn neurons of vehicle-treated mice after filament detachment (Figure 4C, day 14, 2.80 ± 0.23). PTX-treated mice exhibited sustained and intense firing in their superficial dorsal horn neurons upon stimulation with mechanical stimuli (Figure 4C, day 14, 11.18 ± 0.58). Repeated administration of K/Na citrate (2 g/kg/day, p.o.) also suppressed the elevated mechanical stimuli-induced firing in PTX-treated mice by 57% (Figure 4C, day 14, 4.76 ± 0.40, *p* < 0.0001).

### 2.5. The Preventive Effect of Repeated Oral Administration of K/Na Citrate on PTX-Induced Mechanical Allodynia and Plasma Citric Acid Levels in Rats

**Figure 5:**
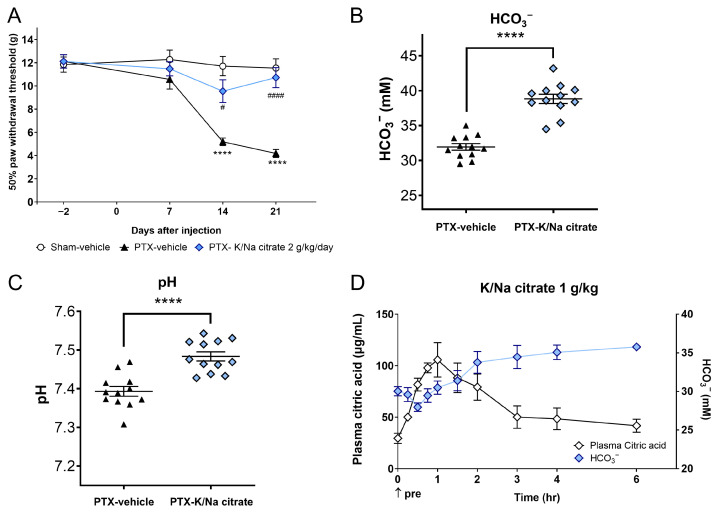
Preventive effect of repeated oral administration of K/Na citrate on PTX-induced mechanical allodynia and plasma citric acid levels in rats. (**A**) Time course of paw withdrawal threshold in the PTX-induced CIPN rat model with or without K/Na citrate treatment (2 g/kg/day, p.o.). Data are presented as the mean ± SEM (*n* = 12). **** *p* < 0.0001 vs. sham-vehicle. ^#^ *p* < 0.05, ^####^ *p* < 0.0001 vs. PTX-vehicle (two-way repeated measures ANOVA with post hoc Tukey–Kramer multiple comparison tests). (**B**,**C**) Blood HCO_3_^−^ concentrations (**B**) and pH (**C**) in rats from each group in (**A**), measured on day 21 at 3 h after the administration of K/Na citrate. Data are presented as mean ± SEM (*n* = 12). **** *p* < 0.0001 vs. PTX-vehicle (Student’s *t*-test). (**D**) Changes in the plasma concentrations of citric acid and HCO_3_^−^ in normal Wistar rats after administration of K/Na citrate (1 g/kg, p.o.) only. Data are presented as mean ± SEM (*n* = 3).

We investigated whether K/Na citrate could alleviate the allodynia induced by repeated PTX injection in rats. Mechanical allodynia was induced by administering PTX (2 mg/kg, i.p.) on four alternate days (days 0, 2, 4, and 6) in rats, resulting in a gradual decrease in the withdrawal threshold of the hind paw to mechanical stimulation on day 14 (5.18 ± 0.33 g, PTX-vehicle group; 11.71 ± 0.83 g, sham-vehicle group, *p* < 0.0001) and day 21 (4.18 ± 0.34 g, PTX-vehicle; 11.53 ± 0.81 g, sham-vehicle group, *p* < 0.0001) (Figure 5A). K/Na citrate (2 g/kg/day, p.o.) was administered twice daily from days −2 to 22, leading to a significant suppression of mechanical allodynia on days 14 (9.55 ± 0.98 g, PTX-K/Na citrate group, *p* < 0.05) and 21 (10.77 ± 0.86 g, PTX-K/Na citrate group, *p* < 0.0001) (Figure 5A). In the PTX-induced CIPN rats, the repeated administration of K/Na citrate significantly increased blood HCO_3_^−^ concentrations (Figure 5B, 38.8 ± 0.7, PTX- K/Na citrate; 32.0 ± 0.5, PTX-vehicle group, *p* < 0.0001) and blood pH (Figure 5C, 7.48 ± 0.01, PTX- K/Na citrate group; 7.39 ± 0.01, PTX-vehicle group, *p* < 0.0001) on day 21 at 3 h post administration; this is consistent with the effects observed in PTX-induced CIPN mice.

Furthermore, we assessed the impact of a single dose of K/Na citrate on plasma citrate and HCO_3_^−^ levels. The plasma citrate level peaked 1 h after K/Na citrate administration and returned to baseline levels approximately 3 h after K/Na citrate administration. In contrast, the plasma HCO_3_^−^ level started to increase 2 h after the administration and remained elevated up to 6 h later (Figure 5B).

### 2.6. Effect of Repeated Oral Administration of K/Na Citrate on Plasma Complement Levels

**Figure 6:**
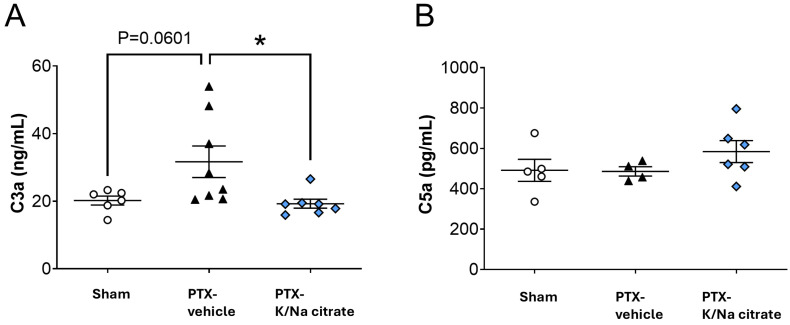
Effect of repeated oral administration of K/Na citrate on the concentrations of complement component anaphylatoxins C3a and C5a in rat plasma. (**A**) C3a and (**B**) C5a rat plasma complement component levels on day 22 in rat PTX-treated model measured using an enzyme-linked immunosorbent assay (ELISA) kit. Data are presented as mean ± SEM (*n* = 4–8). * *p* < 0.05 (one-way ANOVA with post hoc Tukey–Kramer multiple comparison tests).

The activation of complement components C3a and C5a, which have been reported to be associated with PTX-induced peripheral neuropathy [27,28,29], was assessed in PTX-treated rats. The rats were given repeated injections of PTX (2 mg/kg, i.p.) or vehicle on four alternate days (days 0, 2, 4, and 6). K/Na citrate (2 g/kg/day, p.o.) or vehicle was administered twice daily from days −2 to 22. On day 22, the PTX injection led to a nonsignificant increase in plasma C3a levels (Figure 6A, 23.2 ± 3.2 ng/mL, sham-vehicle group; 31.7 ± 4.7 ng/mL, PTX-vehicle group, *p* = 0.0601). The prophylactic administration of K/Na citrate significantly decreased plasma C3a levels (Figure 6A, 21.9 ± 2.9 ng/mL, PTX-K/Na citrate group, *p* < 0.05). Plasma C5a levels were not affected by either the PTX or K/Na citrate treatment (Figure 6B, 491.5 ± 54.4 pg/mL, sham-vehicle group; 486.1 ± 23.1 pg/mL, PTX-vehicle group; 584.4 ± 54.6 pg/mL, PTX-K/Na citrate group).

### 2.7. Na Citrate Prevented the Reduction in Neurite Outgrowth in PTX-Exposed Primary Dorsal Root Ganglion (DRG) Neurons

**Figure 7:**
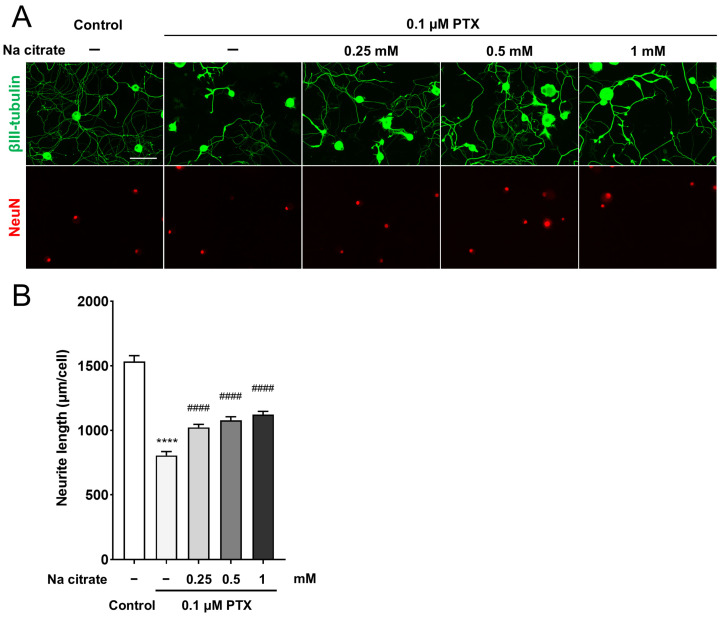
Effect of Na citrate on neurite outgrowth of primary adult rat DRG neurons. (**A**) Representative images of βIII-tubulin (green) and NeuN (red) immunostaining of adult rat DRG neuron cultures exposed to 0.1 µM PTX for 24 h in the presence or absence of Na citrate. Scale bar: 100 µm. (**B**) Total neurite outgrowth in cultures with each treatment. Data are presented as mean ± SEM (*n* = 14 replicate cultures). **** *p* < 0.0001 vs. control, ^####^ *p* < 0.0001 vs. 0.1 μM PTX (one-way ANOVA with post hoc Tukey–Kramer multiple comparison tests).

We investigated the direct effect of Na citrate on axonal integrity in primary cultured adult rat DRG neurons exposed to PTX; the 0.1 μM PTX treatment caused a 48% decrease in neurite outgrowth from DRG neurons compared with untreated neurons (Figure 7A,B, 804.7 ± 30.9 µm, PTX-treated group; 1534.3 ± 44.3 µm, untreated group, *p* < 0.0001). Pretreatment with Na citrate at different concentrations significantly prevented the reduction in the neurite outgrowth of PTX-treated DRG neurons (Figure 7A,B, 1022.2 ± 24.3, 1077.4 ± 28.0, and 1122.5 ± 24.6 µm for 0.25, 0.5, and 1 mM of Na citrate, respectively, *p* < 0.0001). This result indicates that Na citrate exerts a direct protective effect on DRG neurons stressed by PTX exposure.

### 2.8. Therapeutic Effect of Repeated Oral Administration of K/Na Citrate on PTX-Induced Mechanical Allodynia

**Figure 8:**
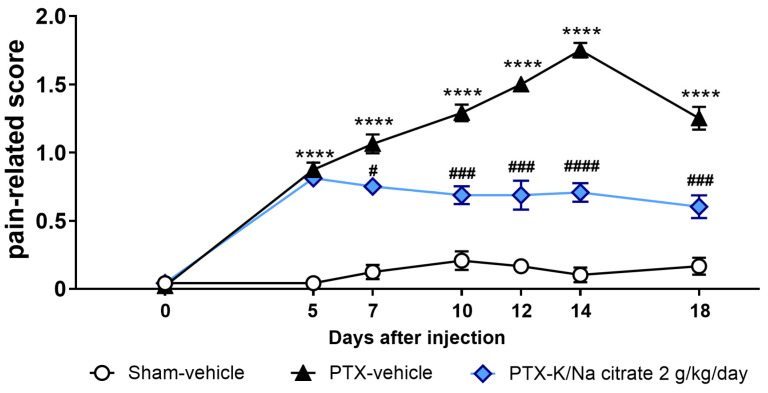
Therapeutic effect of repeated oral administration of K/Na citrate on PTX-induced mechanical allodynia in mice. Time course of the pain-related scores in response to mechanical stimulus in PTX-treated mice. The mice were administered K/Na citrate from days 5 to 18. Data are presented as mean ± SEM (*n* = 8). **** *p* < 0.0001 vs. sham-vehicle. ^#^ *p* < 0.05, ^###^ *p* < 0.001, ^####^ *p* < 0.0001 vs. PTX-vehicle (two-way repeated measures ANOVA with post hoc Holm–Sidak multiple comparison test).

We also investigated the potential therapeutic effects of K/Na citrate on mechanical allodynia by administering K/Na citrate after the development of PTX-induced mechanical allodynia in mice. Mice were given a single injection of PTX (5 mg/kg, i.p.) or vehicle on day 0. K/Na citrate (2 g/kg/day, p.o.) or vehicle was administered twice daily from days 5 to 18. Consistent with previous findings, the PTX treatment led to an increase in the pain-related scores on day 5 (Figure 8, 0.9 ± 0.1, PTX-vehicle group; 0.0 ± 0.0, sham-vehicle group, *p* < 0.0001). When K/Na citrate administration commenced on day 5, the PTX-vehicle group showed a continued increase in their pain-related scores until day 14, whereas the PTX-K/Na citrate group maintained pain-related scores similar to those on day 5 (Figure 8, day 14, 1.8 ± 0.1, PTX-vehicle group; 0.7 ± 0.1, PTX-K/Na citrate group, *p* < 0.05).

## 3. Discussion

In this study, the alkalization of blood or extracellular fluid was demonstrated to be effective in inhibiting PTX-induced peripheral neuropathy. Our study using murine and rat models revealed that the repeated prophylactic administration of K/Na citrate can prevent the development of PTX-induced mechanical allodynia (Figure 1 and Figure 5). Additionally, K/Na citrate was able to prevent the PTX-induced exacerbation of mechanical allodynia, even when treatment was started immediately after the onset of allodynia (Figure 8).

K/Na citrate, which contains Na and K citrate as its active ingredients, has been used in the treatment of metabolic acidosis [30]. Citrate is metabolized to HCO_3_^−^ mainly in the liver after oral administration [31,32,33,34,35]. NaHCO_3_ provides HCO_3_^−^ by dissociating into Na^+^ and HCO_3_^−^ in the body. The HCO_3_^−^ buffering system plays a crucial role in maintaining pH homeostasis in the bloodstream by regulating the balance of carbonic acid (H_2_CO_3_), HCO_3_^−^, and carbon dioxide (CO_2_), thereby contributing to the overall regulation of pH in the body.

In this study, K/Na citrate and NaHCO_3_ were used to alkalinize blood pH. Both compounds attenuated PTX-induced mechanical allodynia, with K/Na citrate exhibiting a stronger effect against mechanical allodynia (Figure 3). This suggests that mechanisms other than alkalinization may contribute to the observed effects. The distinct modes of action in the alkalinizing achieved by K/Na citrate and NaHCO_3_ imply that citrate, before metabolizing into HCO_3_^−^, may possess inherent properties that allow it to attenuate mechanical allodynia.

To better understand the mechanisms of CIPN improvement involved in this alkalinizing effect, we investigated its impact on the immune system. Studies have shown that the immune system and immune-mediated neuroinflammation play a critical role in the development of CIPN, including that induced by PTX [36,37,38,39,40]. Additionally, studies suggest that a complement, a key component of the innate immune system, is involved in the pathogenesis of PTX-induced CIPN [27,28,29,41].

The activation of complements through the classical, lectin, or alternative pathways leads to the activation of the complement components C3 and C5, which are central components of the complement system, resulting in the formation of a membrane attack complex that can cause direct cell damage or lysis.

K/Na citrate reduced plasma C3a levels in a PTX-induced CIPN rat model (Figure 6). It has been reported that C3a and C5a, produced through complement activation, can promote inflammation and potentially mediate neuropathic pain [42]. In our study, the plasma C3a levels in the PTX-induced CIPN rat model exhibited a tendency to increase, indicating an inflammatory response. However, treatment with K/Na citrate appeared to suppress this increase in C3a levels, indicating a potential mechanism for inhibiting the development of mechanical allodynia.

Complement activation is known to be influenced by pH, with both animal and clinical studies showing that hypoxia and reperfusion can lead to complement activation [43,44,45,46]. Furthermore, a low pH has been found to activate alternative pathways [47], and in acidotic and hypoxic conditions, the complement system is activated, resulting in the increased production of C3a and C5a [43,48]. The reduced peripheral blood flow velocity caused by PTX and neuroinflammation involving immune cells like microglia, astrocytes, and macrophages may cause a local pH reduction in peripheral nerves [14,15,16,18]. Therefore, we hypothesize that alkalization plays a role in inhibiting PTX-induced mechanical allodynia through K/Na citrate.

K/Na citrate is presumed to involve citrate’s alkalizing properties; hence, we focused on its potential neuroprotective effects. Previous studies have shown that PTX can induce neurotoxicity, resulting in demyelination, axonal degeneration, impaired axonal trafficking, direct injury to peripheral nerves, and a loss of nerve fibers [49,50,51,52]. Among these neurotoxic effects, a decrease in intraepidermal nerve fibers has been linked to the development of peripheral neuropathy [53]. In this study, we investigated the impact of Na citrate on neurite outgrowth in primary DRG cells as a measure of neurotoxicity. Our results revealed that pretreatment with Na citrate effectively prevented PTX-induced reductions in neurite outgrowth in a dose-dependent manner (Figure 7). These findings suggest that Na citrate helps mitigate the neurotoxic effects of PTX.

In this study, we recorded the activity of superficial dorsal horn neurons using extracellular electrophysiological recording methods. Previous findings indicated that mechanical stimulation increased the firing and spontaneous firing of superficial dorsal horn neurons in PTX-treated mice, resembling mechanical allodynia [54]. Interestingly, the prophylactic administration of K/Na citrate suppressed both mechanically stimulated firing and spontaneous firing (Figure 4). This indicates that inhibiting the input of abnormal stimuli into the dorsal horn of the spinal cord, which is the input site for the peripheral communication of information, may preserve the normal ascending transmission of pain sensory signals from the periphery through the inhibition of complement activity and the neuroprotective action of K/Na citrate.

Furthermore, we confirmed that the repeated administration of K/Na citrate does not inhibit the anticancer effects of PTX in pancreatic tumor-bearing mice (unpublished data). In addition, K/Na citrate was found to inhibit the exacerbation of CIPN even when administered at the early stage of pain onset (Figure 8). Therefore, the clinical application of K/Na citrate is expected to offer a valuable therapeutic approach that can manage the onset of CIPN without compromising the anticancer effects of PTX.

This study suggests that K/Na citrate is beneficial in preventing and alleviating PTX-induced peripheral neuropathy, potentially through its alkalinizing effect and citrate properties. However, the specific contributions of these mechanisms to CIPN improvement and their underlying mechanisms require further investigation.

In this study, we demonstrated that K/Na citrate can suppress the increase in plasma C3a concentration. However, the specific effects of Na/K citrate on the complement cascade and ion channels (such as transient receptor potential channels and Na^+^ channels) associated with the development of CIPN, which have been shown to be upregulated or enhanced by complement activation [28,55], are not fully understood. Further investigations are needed to elucidate these mechanisms. Additionally, our study revealed the protective effect of citrate against PTX neurotoxicity in vitro using primary DRG cells. Future studies are warranted to assess whether K/Na citrate can improve histopathological changes, such as demyelination and axonal degeneration, in animal models of PTX-induced neuropathy.

## 4. Materials and Methods

### 4.1. Animals

#### 4.1.1. Mice

Male C57BL/6 mice weighing 20–26 g at 6 weeks of age were obtained from Japan SLC, Inc. (Shizuoka, Japan) and The Jackson Laboratory Japan, Inc. (Kanagawa, Japan). The mice were housed in a controlled environment with a temperature of 21–23 °C and humidity of 30–62%. The room was lit from 07:00 to 19:00, and food and water were provided ad libitum. In total, 146 mice were used: 112 for 4 behavioral studies (*n* = 8 per group), 15 for the electrophysiological study (*n* = 5 per group), and 19 for assessing blood HCO_3_^−^ concentrations and pH (*n* = 3 or 4 per treatment). One mouse was excluded from the behavioral study because it died due to excessive fighting (Figure 1, PTX-Na/K citrate-2 g/kg/day group). All animal procedures were approved by the Committee for Animal Experiments at the University of Toyama.

#### 4.1.2. Rats

Male Sprague–Dawley rats weighing 250–390 g at 7 weeks of age were obtained from The Jackson Laboratory Japan, Inc. (Kanagawa, Japan). The rats were housed in a controlled environment with a temperature of 22.7–24.1 °C and humidity of 45–65%. The room was lit from 07:00 to 19:00, and food and water were provided ad libitum. A total of 36 rats were used for the behavioral study followed by plasma sampling for analyzing complement components (*n* = 12 per group). All animal procedures were approved by the Committee for Animal Experiments at the Biological Science Center, KAC Co., Ltd. (Shiga, Japan).

Male Sprague–Dawley rats weighing 180–250 g at 5–7 weeks of age and male Wistar rats weighing 180–210 g at 6–7 weeks of age were obtained from The Jackson Laboratory Japan, Inc. (Kanagawa, Japan). The animals were housed in a controlled environment with a temperature of 21–25 °C and humidity of 40–70%. The room was lit from 07:00 to 19:00, and food and water were provided ad libitum. Two Sprague–Dawley rats were used for the primary culture of DRG neurons, and three Wistar rats were used for assessing plasma concentrations of citric acid and HCO_3_^−^. All animal procedures were approved by the Committee for Animal Experiments at Discovery Research Laboratories, Nippon Chemiphar Co., Ltd. (Saitama, Japan).

### 4.2. Test Compound

K/Na citrate (Uralyt-U Combination Powder, Nippon Chemiphar Co., Ltd., Tokyo, Japan), a mixture composed of 2 mol potassium citrate and 2 mol sodium citrate or NaHCO_3_ (Tokyo Chemical Industry Co., Ltd., Tokyo, Japan), was dissolved in distilled water (Otsuka Pharmaceutical Factory, Inc., Tokushima, Japan).

### 4.3. Animal Models

#### 4.3.1. Mouse Study

PTX (FUJIFILM Wako Pure Chemical Corporation, Osaka, Japan) was dissolved in a vehicle that consisted of saline (Otsuka Pharmaceutical Factory, Inc., Tokushima, Japan) containing 10% (*v*/*v*) Kolliphor (C5135, Sigma-Aldrich, Inc., St. Louis, MO, USA) and 10% (*v*/*v*) ethanol. PTX was administered i.p. at a dose of 5 mg/kg [18] on day 0. K/Na citrate or NaHCO_3_ was administered p.o. twice daily at doses of 2 g/kg/day or 1.5 g/kg/day, respectively, starting from 2 days before PTX administration until the end of the study. However, in testing for the therapeutic effect of K/Na citrate on PTX-induced mechanical allodynia, K/Na citrate was administered p.o. at a dose of 2 g/kg/day twice daily from days 5 to 18.

#### 4.3.2. Rat Study

Taxol (PTX, Bristol-Myers Squibb Company, Princeton, NJ, USA) was reconstituted in a saline vehicle. Taxol was administered i.p. at a dose of 2 mg/kg every other day (4 times), starting from day 0. K/Na citrate, at 2 g/kg/day, or vehicle was administered p.o. twice daily from days −2 to 22.

### 4.4. Behavioral Experiments

#### 4.4.1. Mouse Study

Mice were individually placed in plastic cages (11 cm × 18 cm × 15 cm) with a wire mesh bottom and allowed to acclimatize for at least 30 min. Mechanical allodynia in the hind paw was assessed using a fine vFF with a bending force of 0.69 mN (North Coast Medical Inc., Morgan Hill, CA, USA) [54,56]. The vFF was pressed perpendicularly against the central part of the plantar hind paw and held for 1–3 s with slight pressure. Responses to the stimulus were scored as follows: no reaction (0), lifting of the hind paw (1), and licking and flinching of the hind paw (2). The stimulation was repeated three times on each hind paw with intervals of several seconds and at the same intensity, and the average value of six trials was used as the response score (maximum score of 2).

#### 4.4.2. Rat Study

Rats were placed in individual cages. Mechanical allodynia in the hind paw was assessed using a fine vFF and bending forces of 0.4, 0.6, 1, 2, 4, 6, 8, and 15 g. The vFF was pressed perpendicularly against the central part of the plantar hind paw for 3–4 s, and the avoidance response (e.g., foot retraction) was recorded. The 50% paw withdrawal threshold was calculated using the up–down method [57].

### 4.5. Electrophysiological Recording

The mice were anesthetized with urethane (1.5 g/kg, i.p.), which provides long-lasting and steady anesthesia without the need for additional doses in most cases. A thoracolumbar laminectomy was performed to expose the L1–L6 vertebrae, after which the animal was placed in a stereotaxic apparatus. Subsequently, the dura mater was removed, and the arachnoid membrane was incised to create a large window for a tungsten microelectrode. The spinal cord surface was irrigated with Krebs solution equilibrated with 95% O_2_ and 5% CO_2_ at a flow rate of 10–15 mL/min, containing 117 mM NaCl, 3.6 mM KCl, 2.5 mM CaCl_2_, 1.2 mM MgCl_2_, 1.2 mM NaH_2_PO_4_, 11 mM glucose, and 25 mM NaHCO_3_ at 37 ± 1 °C. Extracellular single-unit recordings of superficial dorsal horn (lamina I and II) neurons were conducted as previously described [58,59,60,61] at a depth of 20–150 μm from the surface. The recordings were obtained from the superficial dorsal horn neurons in slices from the same spinal level of same-age mice. Unit signals were amplified using an EX1 amplifier (Dagan Corporation, Minneapolis, MN, USA), digitized with an analog-to-digital converter (Digidata 1400A, Molecular Devices, LLC., San Jose, CA, USA), and stored on a personal computer using a data acquisition program (Clampex software, version 10.2, Molecular Devices, LLC., San Jose, CA, USA). The skin areas where touch (with a cotton wisp) or noxious pinch (with forceps) stimuli elicited a neural response were identified. Mechanical stimuli were applied to the skin folding of the ipsilateral hind limb using a fine vFF with a bending force of 0.69 mN for 5 s at the maximal response point of each mouse’s respective receptive area.

### 4.6. Measurement of Plasma Citric Acid, Blood HCO_3_^−^, and pH

C57BL/6 mice were orally administered K/Na citrate (1 g/kg) under non-fasting conditions. Blood samples were collected from the tail vein of the mice at predose, 1.5, 3, 5.5, and 8 h after oral administration. In PTX-treated mice, blood samples were collected from the tail vein of the mice 2 h after administering K/Na citrate on day 14 after PTX treatment.

Wistar rats were orally administered K/Na citrate (1 g/kg) under fasting conditions. Blood samples were collected from the tail vein of the rats at predose, 0.25, 0.5, 0.75, 1, 1.5, 2, 3, 4, and 6 h after oral administration. In the PTX-induced CIPN rat models, blood samples were collected from the tail vein of the rat 3 h after administering K/Na citrate on day 21 after PTX treatment.

In the above experiment, blood samples were drawn using lithium heparin as an anticoagulant. Blood pH and HCO_3_^−^ concentrations were measured using the i-STAT 1 analyzer (Abbott Laboratories, Abbott Park, IL, USA). Plasma samples were obtained by centrifugation of the blood (1500× *g*, 4 °C, 15 min) and were used for citric acid measurements using a Citrate Assay Kit (Sigma-Aldrich, Inc., St. Louis, MO, USA).

### 4.7. Preventive and Dose-Dependent Effect of K/Na Citrate on Mechanical Allodynia

In the mouse study, mechanical allodynia was induced by a single injection of PTX at 5 mg/kg. K/Na citrate was administered p.o. at doses of 0.25, 0.5, and 1 g/kg twice daily, starting 2 days before PTX injection until the end of the study. Blood HCO_3_^−^ levels were measured 2 h after K/Na citrate administration on day 14 after PTX treatment.

In the rat study, mechanical allodynia was induced by repeated injections of PTX (2 mg/kg, i.p.) on four alternate days (days 0, 2, 4, and 6). K/Na citrate was administered p.o. at a dose of 1 g/kg twice daily, starting 2 days before the first PTX injection until the end of the study.

### 4.8. Therapeutic Effect of K/Na Citrate on Mechanical Allodynia in PTX-Treated Mice

PTX-induced mechanical allodynia was established using the abovementioned method (see Section 4.4.1). K/Na citrate was administered p.o. at a dose of 1 g/kg twice daily starting from 5 days after the PTX injection until the end of the study.

### 4.9. Effect of Repeated Oral Administration of K/Na Citrate on the Concentrations of Complement Components, Anaphylatoxins C3a and C5a, in Rat Plasma

Rats were given four injections of PTX (2 mg/kg, i.p., every other day) or vehicle on day 0. K/Na citrate (2 g/kg/day, p.o.) or vehicle was administered twice daily from days −2 to 22. Plasma samples were collected from the rats’ jugular veins using lithium heparin as an anticoagulant on day 22. Plasma C3a and C5a levels were measured using the Rat Complement Fragment 3a (C3a) ELISA Kit (CSB-E08510r, Cusabio Technology LLC, Houston, TX, USA) and the Rat Complement C5a ELISA Kit (LS-F34383-1, Lifespan Biosciences, Inc., Seattle, WA, USA), respectively, following the manufacturer’s instructions.

### 4.10. Assessment of Neurite Outgrowth in Primary Adult Rat DRG Cultures

Based on previous studies [62,63,64], DRG neurons from 5–7-week-old Sprague–Dawley rats were dissociated. Briefly, bilateral thoracic and lumbar DRG were isolated and treated with 1.25 mg/mL of collagenase A (10103578001, Roche, Mannheim, Germany) and 2.5 mg/mL of dispase II (D4693, Sigma-Aldrich, Inc., St. Louis, MO, USA) in Hank’s Balanced Salt Solution without calcium and magnesium (14175095, Thermo Fisher Scientific, Inc., Waltham, MA, USA) for 90 min. Then, they were treated with 0.25% trypsin-ethylenediaminetetraacetic acid (25200056, Thermo Fisher Scientific, Inc., Waltham, MA, USA) for 3 min. The digest was neutralized using Dulbecco’s Modified Eagle Medium (11885084, Thermo Fisher Scientific, Inc., Waltham, MA, USA) containing 10% fetal bovine serum (S1600, Biowest, Nuaillé, France) and 0.1% penicillin–streptomycin (15140122, Thermo Fisher Scientific, Inc., Waltham, MA, USA) and mechanically dissociated using a pipette and 23- and 28-gauge needles. The resulting cell suspension was layered on a 30%/60% Percoll (17089102, Global Life Sciences Technologies Japan K.K., Tokyo, Japan) gradient and centrifuged for 15 min at 1000× *g* at 4 °C, and the cell fraction between the 30% and 60% interface was collected. The pellet was resuspended into Neurobasal A medium (10888022, Thermo Fisher Scientific, Inc., Waltham, MA, USA) with 2% B-27 supplement without antioxidants (10889038, Thermo Fisher Scientific, Inc., Waltham, MA, USA), GlutaMAX supplement (35050061, Thermo Fisher Scientific, Inc., Waltham, MA, USA), 0.1% penicillin–streptomycin, 0.1 ng/mL nerve growth factor (13257-019, Thermo Fisher Scientific, Inc.), 1 ng/mL glial cell line-derived neurotrophic factor, and 2’-deoxy-5-fluorouridine (D2235, Tokyo Chemical Industry Co., Ltd., Tokyo, Japan). The cells were plated on 96-well glass-bottom plates which were previously coated with 0.1 mg/mL poly-D-lysine (P6407, Sigma-Aldrich, Inc., St. Louis, MO, USA) and 10 µg/mL laminin (L2020, Sigma-Aldrich, Inc., St. Louis, MO, USA) at a density of 1000 neurons per well. The following day, the cells were pretreated with Na citrate for 4 h and exposed to PTX (0.1 μM) for 24 h. After treatment, the cells were fixed with ice-cold methanol for 15 min at −30 °C, washed thrice with phosphate-buffered saline (05913, Shimazu Corporation, Tokyo, Japan), blocked with 1% bovine serum albumin (B4287, Sigma-Aldrich, Inc., St. Louis, MO, USA) for 1 h at room temperature, immunostained with anti-βIII-tubulin antibody (1:2000, G712A, Promega Corporation, Madison, WI, USA) and anti-NeuN antibody (1:800, 24307, Cell Signaling Technology, Inc., Danvers, MA, USA) overnight at 4 °C, and exposed to Alexa Fluor 488-conjugated antimouse IgG (1:1000, A-11001, Thermo Fisher Scientific, Inc., Waltham, MA, USA) and Alexa Fluor 594-conjugated anti-rabbit IgG (1:1000, A-11012, Thermo Fisher Scientific, Inc., Waltham, MA, USA) for 1 h at room temperature. Images were captured using a BZ-X810 microscope (Keyence Corporation, Osaka, Japan) with an original image size of 960 × 720 pixels and cropped to 672 × 503 pixels using a BZ-X800 Image Converter 1.1.2.4 software (Keyence Corporation, Osaka, Japan) to trim overlapping parts. Images for βIII-tubulin were subjected to haze reduction using the BZ-X800 analyzer 1.1.2.4 software (Keyence Corporation, Osaka, Japan). The total neurite length, in pixels, was quantified using ImageJ 1.54f software (National Institute of Health, Bethesda, MD, USA) with the NeurphologyJ plugin [65]. Soma detection was performed using ImageJ 1.54f software on NeuN images. The total neurite length in pixels was converted to that in µm and adjusted for the total soma count.

### 4.11. Statistical Analysis

Statistical analyses were conducted using GraphPad Prism 10.3.0 software (GraphPad Software, Inc., San Diego, CA, USA). Data were presented as the mean and SEM. Statistical significance was assessed using Student’s *t*-test (Figure 2C,D and Figure 5B,D) or a one-way ANOVA followed by a post hoc Holm–Sidak test (Figure 4A–C) or Tukey–Kramer test (Figure 6A,B, and Figure 7) or two-way repeated measures ANOVA followed by a post hoc Holm–Sidak test (Figure 1A–C, Figure 2A,B, Figure 3 and Figure 8) or Tukey–Kramer test (Figure 5A). A *p*-value of less than 0.05 was considered statistically significant. Outliers were identified and eliminated using the Smirnov–Grubbs test (Figure 6A,B), a method for outlier detection that assumes a normal distribution of the data.

## 5. Conclusions

Our findings suggest that K/Na citrate may be useful in the prevention and treatment of PTX-induced peripheral neuropathy.

## Data Availability

The data presented in this study are available on request from the corresponding author.

## Abbreviations

The following abbreviations are used in this manuscript:

ANOVA	Analysis of variance
CIPN	Chemotherapy-induced peripheral neuropathy
DRG	Dorsal root ganglion
ELISA	Enzyme-linked immunosorbent assay
i.p.	Intraperitoneal
p.o.	Per os
SEM	Standard error of the mean
vFF	von Frey filament
